# Prediction of 2-Year Cognitive Outcomes in Very Preterm Infants Using Machine Learning Methods

**DOI:** 10.1001/jamanetworkopen.2023.49111

**Published:** 2023-12-26

**Authors:** Andrea K. Bowe, Gordon Lightbody, Anthony Staines, Deirdre M. Murray, Mikael Norman

**Affiliations:** 1 INFANT Research Centre, University College Cork, Cork, Ireland; 2 Department of Electrical and Electronic Engineering, University College Cork, Cork, Ireland; 3 School of Nursing, Psychotherapy, and Community Health, Dublin City University, Dublin, Ireland; 4 Department of Paediatrics, Cork University Hospital, Cork, Ireland; 5 Department of Clinical Science, Intervention, and Technology, Karolinska University Hospital, Karolinska Institutet, Stockholm, Sweden; 6 Department of Neonatal Medicine, Karolinska University Hospital, Karolinska Institutet, Stockholm, Sweden

## Abstract

**Importance:**

Early intervention can improve cognitive outcomes for very preterm infants but is resource intensive. Identifying those who need early intervention most is important.

**Objective:**

To evaluate a model for use in very preterm infants to predict cognitive delay at 2 years of age using routinely available clinical and sociodemographic data.

**Design, Setting, and Participants:**

This prognostic study was based on the Swedish Neonatal Quality Register. Nationwide coverage of neonatal data was reached in 2011, and registration of follow-up data opened on January 1, 2015, with inclusion ending on September 31, 2022. A variety of machine learning models were trained and tested to predict cognitive delay. Surviving infants from neonatal units in Sweden with a gestational age younger than 32 weeks and complete data for the Bayley Scales of Infant and Toddler Development, Third Edition cognitive index or cognitive scale scores at 2 years of corrected age were assessed. Infants with major congenital anomalies were excluded.

**Exposures:**

A total of 90 variables (containing sociodemographic and clinical information on conditions, investigations, and treatments initiated during pregnancy, delivery, and neonatal unit admission) were examined for predictability.

**Main Outcomes and Measures:**

The main outcome was cognitive function at 2 years, categorized as screening positive for cognitive delay (cognitive index score <90) or exhibiting typical cognitive development (score ≥90).

**Results:**

A total of 1062 children (median [IQR] birth weight, 880 [720-1100] g; 566 [53.3%] male) were included in the modeling process, of whom 231 (21.8%) had cognitive delay. A logistic regression model containing 26 predictive features achieved an area under the receiver operating curve of 0.77 (95% CI, 0.71-0.83). The 5 most important features for cognitive delay were non-Scandinavian family language, prolonged duration of hospitalization, low birth weight, discharge to other destination than home, and the infant not receiving breastmilk on discharge. At discharge from the neonatal unit, the full model could correctly identify 605 of 650 infants who would have cognitive delay at 24 months (sensitivity, 0.93) and 1081 of 2350 who would not (specificity, 0.46).

**Conclusions and Relevance:**

The findings of this study suggest that predictive modeling in neonatal care could enable early and targeted intervention for very preterm infants most at risk for developing cognitive impairment.

## Key Points

**Question:**

Can easily available neonatal data identify very preterm infants who will exhibit cognitive delay later in life?

**Findings:**

In this prognostic study of cognitive outcomes at 2-year follow-up among 1062 infants born very preterm, a logistic regression model containing 26 neonatal features identified 93% of very preterm infants who screened positive for cognitive delay at 2-year follow up, with a specificity of 46%.

**Meaning:**

Use of this model could target those very preterm infants at the highest risk of cognitive delay to receive early and effective intervention.

## Teaser

This prognostic study of very preterm infants assesses the use of an explainable algorithm to predict cognitive delay in infants at 2 years of age.

## Introduction

Each year, 2.2 million infants are born very preterm (VPT), that is, at a gestational age (GA) younger than 32 weeks.^1^ Accompanying improved survival, more children are at risk for long-term impairments associated with prematurity, such as cognitive difficulties.^2,3,4,5^ The mean IQ, across childhood, for VPT infants has been reported to be almost 1 SD lower than their full-term counterparts, equivalent to a mean difference of approximately 13 IQ points.^6^ This finding has important implications for academic achievement, socioeconomic status, and physical and mental health.^7,8,9,10,11,12^ Since 1990, the preterm infant mortality rate in high-income countries has decreased by 4% per year, yet no significant improvements in cognitive outcomes have been achieved.^2,6,13^

The causal pathways underpinning the association between VPT birth and cognitive impairment remain unclear, making it difficult to implement primary prevention strategies.^6^ Secondary prevention, in the form of early intervention, has so far been the only effective way forward.^14^ However, many of the interventions studied were highly resource intensive—commencing in the hospital, followed by frequent visits to the families in their homes, for durations of up to 3 years.^14^ Early and accurate identification of infants most in need of these interventions has therefore become increasingly important.

Biological and environmental factors, as well as clinical events in the perinatal period, contain prognostic information about cognitive function in preterm children younger than 5 years.^15^ Various prognostic models have been reported, but few have been able to fully account for nonlinear relationships and interactions between predictors or have validated their models on unseen data.^16^ The aim of this study was to develop an explainable algorithm for use in VPT infants to predict cognitive delay (CD) at 2 years of age. For this purpose, perinatal characteristics of those who did and did not experience CD at 2 years of age were described. A data-driven approach to identify the relevant predictors of cognitive outcome was then used, and a variety of machine learning models were trained and subsequently tested on an unseen test set. Finally, the most important predictive features were identified.

## Methods

The Swedish Ethical Review Authority approved this prognostic study on March 28, 2022. This authority also waived informed consent from parents or caregivers, who had been informed that perinatal and follow-up data were registered, with a possibility to opt out at any time (used by <5 families in the study period). The Transparent Reporting of a Multivariable Prediction Model for Individual Prognosis or Diagnosis (TRIPOD) reporting guidelines were followed in the reporting of this study.^17^

### Data

Data were from the Swedish Neonatal Quality Register (SNQ). The SNQ is a nationwide, population-based register that captures all infants admitted for neonatal care. The register provides detailed clinical information on conditions, investigations, and treatments initiated during pregnancy, delivery, and neonatal unit admission. Nationwide coverage of neonatal data was reached in 2011, and registration of follow-up data opened on January 1, 2015, with inclusion ending on September 31, 2022. Details on data reporting, completeness and validity of the register have been previously published.^18^

As part of a national follow-up program launched in 2015, high-risk survivors of neonatal intensive care were recommended to receive neurodevelopmental assessment, including the Bayley Scales of Infant and Toddler Development, Third Edition (BSID-III) scheduled at corrected age of 2 years. The primary aim of the follow-up was to screen for different impairments, particularly in those with a GA younger than 28 weeks.^19^ The results of the assessments should be reported to the SNQ.

### Study Population

Eligible for inclusion in this study were VPT 2-year-old children with data for the BSID-III cognitive index or scale score. Children with a cognitive assessment but having a major congenital anomaly as defined by the European Registration of Congenital Anomalies and Twins^20^ (n = 142) were excluded. There were 59 VPT children who attended for BSID-III assessment but could not complete the test (Table 1).

**Table 1.** Features Assessed as Important or Tentative for Future Cognitive Function in Very Preterm Infants (GA <32 Weeks)

Feature	Valid No.	Total (N = 1062)	Typical cognitive development (n = 831)	Cognitive delay (n = 231)	*P* value
**Pregnancy**					
Gestational diabetes, No. (%)	1062	14 (1.3)	8 (1.0)	6 (2.6)	.09^a^
**Birth**					
GA, mean (SD), wk	1062	26.5 (2.2)	26.7 (2.2)	26.0 (2.2)	<.001^b^
Birth weight, median (IQR), g	1055	880 (720 to 1100)	900 (760 to 1125)	800 (660 to 980)	<.001^c^
*z* Score for birth weight, median (IQR)	1056	−1.1 (−2.3 to −0.3)	−1.0 (−2.2 to −0.2)	−1.2 (−2.5 to −0.4)	.16^c^
Head circumference, mean (SD), cm	883	24.7 (2.5)	24.9 (2.5)	24.2 (2.5)	<.001^b^
Sex, No. (%)					
Male	1062	566 (53.3)	414 (49.8)	152 (65.8)	<.001^d^
Female	1062	496 (46.7)	417 (50.2)	79 (34.2)	
Apgar score, median (IQR)					
5 min	1036	8 (6 to 9)	8 (6 to 9)	7 (5 to 9)	.001^c^
10 min	1033	9 (8 to 10)	9 (8 to 10)	9 (7 to 10)	.01^c^
**Neonatal resuscitation**					
Intubation in delivery room, No. (%)	1053	371 (35.2)	265 (32.1)	106 (46.7)	<.001^d^
**Respiratory**					
Bronchopulmonary dysplasia, No. (%)^e^	1062	637 (60.0)	475 (57.2)	162 (70.1)	<.001^d^
Extra oxygen, median (IQR), d	1062	49 (15 to 80)	45 (12 to 74)	67 (34.5 to 98.5)	<.001^c^
Duration of CPAP, median (IQR), d	1038	26 (9 to 39)	25 (8 to 38)	30 (14 to 44)	<.001^c^
Duration of mechanical ventilation, median (IQR), d	1062	4 (0 to 17)	3 (0 to 14)	12 (0 to 26)	<.001^c^
Duration conventional mechanical ventilation, median (IQR), d	1062	1 (0 to 6)	1 (0 to 5)	3 (0 to 9)	<.001^c^
Duration of HFOV, median (IQR), d	1062	0 (0 to 8)	0 (0 to 5)	3 (0 to 15)	<.001^c^
Postnatal steroids (systemic), No. (%)	1062	341 (32.1)	238 (28.6)	103 (44.6)	<.001^d^
**Infection**					
Duration of antibiotics, median (IQR), d	1062	14 (7 to 27)	12 (6 to 25)	20 (8 to 33)	<.001^c^
**Neurology**					
IVH grade, No. (%)					
No confirmed IVH	1045	745 (71.3)	602 (73.5)	143 (63.3)	<.001^d^
1	1045	129 (12.3)	100 (12.2)	29 (12.8)	
2	1045	97 (9.3)	73 (8.9)	24 (10.6)	
3	1045	36 (3.4)	24 (2.9)	12 (5.3)	
4	1045	38 (3.6)	20 (2.4)	18 (8.0)	
Non-IVH intracranial hemorrhage, No. (%)	1062	12 (1.1)	6 (0.7)	6 (2.6)	.03^a^
cPVL, No. (%)	1062	17 (1.6)	8 (1.0)	9 (3.9)	.004^d^
**Other treatments**					
Umbilical artery catheter, No. (%)	1062	861 (81.1)	679 (81.7)	182 (78.8)	.36^d^
Insulin treatment for hyperglycemia, No. (%)	1062	131 (12.3)	88 (10.6)	43 (18.6)	.002^d^
ROP treatment, No. (%)	1000	124 (12.4)	84 (10.8)	40 (17.9)	.007^d^
Duration of inotrope administration, median (IQR), d	1062	0 (0 to 0)	0 (0 to 0)	0 (0 to 1.5)	<.001^c^
No. of plasma transfusions, median (IQR)	1050	2 (1 to 12)	1 (1 to 12)	2 (1 to 20)	<.001^c^
**Hospital stay and discharge**					
Duration of hospital and home care, median (IQR), d	1062	75 (56 to 96)	73 (54 to 94)	85 (65 to 108)	<.001^c^
Duration of hospitalization, median (IQR), d	1062	75 (56 to 96)	72 (53 to 93)	83 (65.5 to 108)	<.001^e^
Discharged to home, No. (%)		927 (87.3)	746 (89.8)	181 (78.4)	<.001^d^
Receiving breast milk on discharge, No. (%)					
None	913	320 (35.0)	235 (32.2)	85 (46.2)	<.001^d^
Partially	913	355 (38.9)	284 (39.0)	71 (38.6)	
Completely	913	238 (26.1)	210 (28.8)	28 (15.2)	
**Sociodemographic characteristics**					
Swedish or other Scandinavian language as the family language, No. (%)	914	641 (70.1)	541 (74.8)	100 (52.4)	<.001^d^
Parental education, No. (%)					
0-3 y	859	8 (0.9)	5 (0.7)	3 (1.6)	<.001^a^
3-6 y	859	3 (0.3)	2 (0.3)	1 (0.5)	
7-9 y	859	50 (5.8)	31 (4.6)	19 (10.4)	
10-12 y	859	322 (37.5)	243 (35.9)	79 (43.2)	
>12 y	859	476 (55.4)	395 (58.4)	81 (44.3)	
**BSID-III^f^**					
Correct age at test, mean (SD), y	1024	2.2 (0.2)	2.2 (0.2)	2.2 (0.2)	.59^b^
Cognition index score, mean (SD)	1062	96.8 (16.1)	103.0 (10.5)	73.5 (10.0)	<.001^b^

Abbreviations: BSID-III, Bayley Scales of Infant and Toddler Development, Third Edition; CPAP, continuous positive airway pressure; cPVL, cystic periventricular leukomalacia; GA, gestational age; HFOV, high-frequency oscillatory ventilation; IVH, intraventricular hemorrhage; ROP, retinopathy of prematurity.

^a^ Fisher exact test.

^b^ Welch 2-sample *t* test.

^c^ Wilcoxon rank sum test.

^d^ Pearson χ^2^ test.

^e^ Defined as a registered *International Statistical Classification of Diseases and Related Health Problems, Tenth Revision* (*ICD-10*) code for bronchopulmonary dysplasia (P27.1) or a registration of supplemental oxygen use at 36 weeks of postmenstrual age.

^f^ Reasons for not completing BSID-III cognitive scale (n = 59) were as follows: child declined or would not participate (n = 23); inattention, hyperactivity, or fatigue (n = 6); language barrier (n = 1); parent declined (n = 4); resources or administrative reason (n = 6); child unable to complete (n = 13); or reason unclear or not recorded (n = 6).

### Outcome

The outcome was cognitive ability at 2-year follow-up, measured using the BSID-III. The BSID-III has not been standardized on a Swedish population. However, in healthy Swedish controls born at term (n = 366) and assessed at 30 months of age, the mean (SD) cognitive index score was 104 (10.6).^21^ On the basis of that study, the SNQ follow-up program applies a threshold score of less than 90 (approximately a −1 SD cutoff) to identify children who may benefit from further investigation or intervention.^19^ In this study, children with a cognitive index score less than 90 were categorized as having CD and those with a score of 90 or greater as having typical cognitive development (TCD).

### Statistical Analysis

#### Descriptive Statistics

The characteristics of participants were compared using Pearson χ^2^ and Fisher exact tests (categorical data), Welch’s 2 sample *t* test (normally distributed continuous data), and Wilcoxon rank sum test (data with nonnormal distribution). Hypothesis tests were 2-tailed, and a priori significance was set at *P* < .05.

#### Data Preparation

Data were handled with the R statistical software package, version 4.1.1 (R Project for Statistical Computing).^22^ A study data set containing 97 potential predictive features, chosen based on SNQ contents, previous literature, plausible hypotheses, and senior author input, was created. Any feature with more than 25% missing values was removed from the data set (eTable 1 in Supplement 1), leaving 90 features categorized as pregnancy; birth; neonatal resuscitation; neonatal respiratory, neurological, infection, or other illnesses; and sociodemographic factors. Remaining missing values (eTable 2 in Supplement 1) were imputed using the missforest package, a random forest imputation method.^23,24^

#### Feature Selection

An “all relevant” approach, aiming to identify all features relevant for the classification, was adopted and the Boruta algorithm was used.^25,26^ For each feature, a corresponding “shadow” feature was created by randomly shuffling the values of the original feature, thereby destroying any possible association with the outcome. A random forest classifier was trained on the original and shadow features. The importance value of each original feature was compared with a threshold—defined as the maximum importance value recorded among the shadow features. If a feature’s importance was higher than this threshold, it was recorded in a vector as a hit. This process was iterated. Features that significantly outperformed the threshold were confirmed important, although those that significantly underperformed were rejected and removed from further iterations. The algorithm stopped when a predefined maximum number of 600 iterations was reached and the remaining features were labeled as tentative.^25,26^

#### Correlated Features

Pearson correlation coefficients were calculated and plotted in a pairwise manner for all features identified as important or tentative. Features with coefficients greater than 0.70 were examined for redundancy. In choosing which features to retain, robustness, dimensionality, correlation with the outcome, expert opinion, and effect on prediction were considered.

#### Modeling

The data set was stratified by the outcome and randomly split into a training set containing 70% of the data and a testing set containing 30%. The outcome classes in the data set were imbalanced, which risked producing a predictive model with a bias toward the majority class. To address this, the synthetic minority oversampling technique (SMOTE) was applied to the training data set.^27,28^

Using all relevant features identified by Boruta, a random forest, logistic regression, support vector machine, and gradient boosting machine algorithm were trained and optimal hyperparameters selected in cross-validated grid search using the caret package (models A-D).^29^ These 4 algorithms were chosen because they have been widely used in health care research, are more likely to be recognized by the clinicians intended to use them, are efficient, and have potential for explainability.^30,31^

#### Evaluation

Internal validation was performed by examining accuracy in 10-fold cross-validation. This process involved splitting the training data set into 10 parts containing equal numbers of observations. At each iteration the model was trained using 90% (9 parts) of the data and validated on the remaining 10%. The procedure was repeated 10 times using a different 10% for validation each time and results were averaged across the folds. The decision threshold was set at a probability of 0.5.

**Figure 1.**
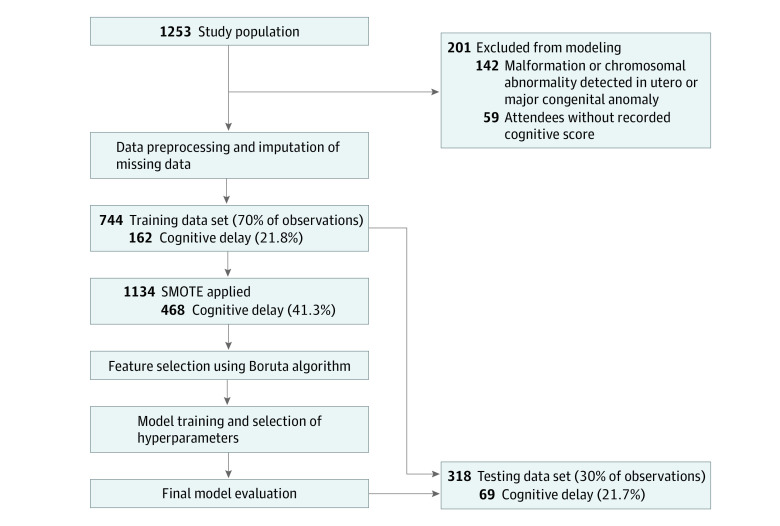
Overview of Modeling Process SMOTE indicates synthetic minority oversampling technique.

The final models with optimally tuned hyperparameters were then externally validated by testing their performance on the unseen test data set. Accuracy, balanced accuracy, sensitivity, and specificity were compared. The area under the receiver operating curve (AUROC) was used to examine performance across all possible decision thresholds.^32^ The modeling process is summarized in Figure 1.

#### Explainability

The explainability of the best-performing models on external validation was explored by examining feature importance and feature effect. Feature importance plots were created using the test data set and the permutation method in the vip package.^33^ Baseline model performance was measured using AUROC. The feature of interest was then randomly shuffled, and model performance was measured again. The difference between the 2 measures was used as a measure of feature importance. For each feature, shuffling was simulated 10 times and importance was averaged across the simulations. Feature effect and more detailed methods are described in the eMethods in Supplement 1.

## Results

### Characteristics of the Study Population

The perinatal, neonatal, and family characteristics of the 1062 children (median [IQR] birth weight, 880 [720-1100] g; 566 [53.3%] male and 496 [46.7%] female) included in the modeling process are shown in Table 1. A description of the cohort across all 90 features included in the selection process is contained in eTable 3 in Supplement 1. A total of 231 children (21.8%) screened positive for CD. The proportion of male children was higher in the CD group than the TCD group (152 [65.8%] vs 414 [49.8%]; *P* < .001). Infants in the CD group had a lower mean (SD) GA (26.0 [2.2] vs 26.7 [2.2] weeks; *P* < .001) and a lower median (IQR) birth weight (800 [660-980] vs 900 [760-1125] g; *P* < .001) than the TCD group. A higher proportion of children with CD than TCD were intubated during neonatal resuscitation (106 [46.7%] vs 265 [32.1%]; *P* < .001). There were also significant differences between the groups in terms of the respiratory complications and oxygenation requirements. The proportion with bronchopulmonary dysplasia was significantly higher in the CD than in the TCD group (162 [70.1%] vs 475 [57.2%]; *P* < .001), as was the proportion requiring postnatal steroids (103 [44.6%] vs 238 [28.6%]; *P* < .001). The median durations of continuous positive airway pressure (CPAP) and conventional or high-frequency oscillatory ventilation were longer for the CD group.

Overall, the incidence of intraventricular hemorrhage (IVH) of any grade was higher (83 [36.7%] vs 217 [26.5%]), and higher grades were more frequently seen, in the CD than in the TCD group. The incidence of non-IVH intracranial hemorrhage was 1.1% (12 of 1062), and the incidence of cystic periventricular leukomalacia was 1.6% (17 of 1062), with a higher incidence in the CD group.

Infants in the CD group had longer mean (SD) neonatal hospital stay (83 [42.5] vs 72 [40.0] days; *P* < .001), and a lower proportion of these infants received any breastmilk at discharge (99 [53.8%] vs 494 [67.8%]). Lower proportions of parents of the CD than the TCD group reported more than 12 years of education (81 [44.3%] vs 395 [58.4%]; *P* < .001) and Scandinavian language as being the family language (100 [52.4%] vs 541 [74.8%]; *P* < .001).

### Training and Testing Data Sets

The original training data set contained 744 infants, of whom 162 (21.8%) screened positive for CD. After SMOTE was applied, the training set consisted of 1134 participants, of whom 468 (41.3%) had CD. The test set was composed of 318 infants, of whom 69 (21.7%) had CD. SMOTE was not applied to the test set.

### Feature Selection

The Boruta algorithm confirmed 27 of 90 features as important, with a further 4 labeled tentative (eFigure 1 in Supplement 1). Among these 31 features, there were 12 features with correlation coefficients greater than 0.70 (eFigure 2 in Supplement 1). Five features were removed to reduce collinearity (Apgar score at 5 minutes, total duration of any mechanical ventilation, duration of supplemental oxygen, duration of hospital and home care combined, and *z* score for birth weight), leaving 26 features detailed in eTable 4 in Supplement 1. Duration of hospitalization and duration of CPAP, as well as birth weight, GA, and head circumference, were highly correlated, but predictive performance was better with all features included. The *z* score for birth weight was removed because this feature was derived from both GA and birth weight and predictive performance was better when the source features were included.

### Model Training and Evaluation

The internal validation results are shown in eTable 5 in Supplement 1. As shown in Table 2 and eFigure 3 in Supplement 1, on the unseen test data, all models achieved an AUROC greater than 0.70. Model D (gradient machine boosting) had an accuracy of 0.77 for identifying infants as having either CD or TCD at 2-year follow-up, with a sensitivity of 0.55 and a specificity of 0.83 at a decision threshold of 0.5. Model B (logistic regression) had an accuracy of 0.76, with a sensitivity of 0.48 and a specificity of 0.84. Model A (random forest) had an accuracy of 0.74, and model C (support vector machine) had an accuracy of 0.70. Although Model D achieved the highest accuracy of 0.77 at the 0.5 threshold, the AUROC curve suggested that model B could achieve the highest sensitivity with alteration of the threshold. Model B, a logistic regression model containing 26 features, achieved an AUROC of 0.77 (95% CI, 0.71-0.83).

**Table 2.** External Validation on Unseen Test Data for Models A to D

Model	Algorithm	No. of features	Hyperparameter final value	Sensitivity	Specificity	AUROC (95% CI)	Balanced accuracy	Accuracy
A	Random forest	26	Mtry (6)	0.49	0.81	0.74 (0.68-0.81)	0.65	0.74
B	Logistic regression	26	NA	0.48	0.84	0.77 (0.71-0.83)	0.66	0.76
C	Support vector machine	26	C(2), Sigma (0.05)	0.64	0.72	0.75 (0.69-0.81)	0.68	0.70
D	Gradient boosting machine	26	Ntrees (800), Interaction depth (15), Shrinkage (0.1), n.minobsinnode (10)	0.55	0.83	0.76 (0.70-0.82)	0.69	0.77

Abbreviations: AUROC, area under the receiver operating characteristic curve; NA, not applicable.

### Application of Model B

The decision threshold of model B was lowered to 0.20 to improve sensitivity. A contingency table showing a hypothetical application is shown in Table 3. Among 3000 VPT infants without major malformations, it would be expected that approximately 650 would have CD at 24 months. At discharge from the neonatal unit, model B could correctly identify 605 of 650 infants who would have CD at 24 months (sensitivity, 0.93), and 1081 of 2350 who would not (specificity, 0.46). Among 1874 infants predicted to have CD, 605 (32.3%) would go on to have it. Among 1126 infants predicted not to have CD, 1081 (96.0%) would not have it.

**Table 3.** Contingency Table for Model B at a Decision Threshold of 0.20^a^

Prediction	Cognitive development at 24 mo		
	Cognitive delay	No cognitive delay	Total
Cognitive delay	605	1269	1874
No cognitive delay	45	1081	1126
Total	650	2350	3000

^a^ Numbers are based on a hypothetical population of 3000 very preterm infants without major malformations.

### Feature Importance

**Figure 2.**
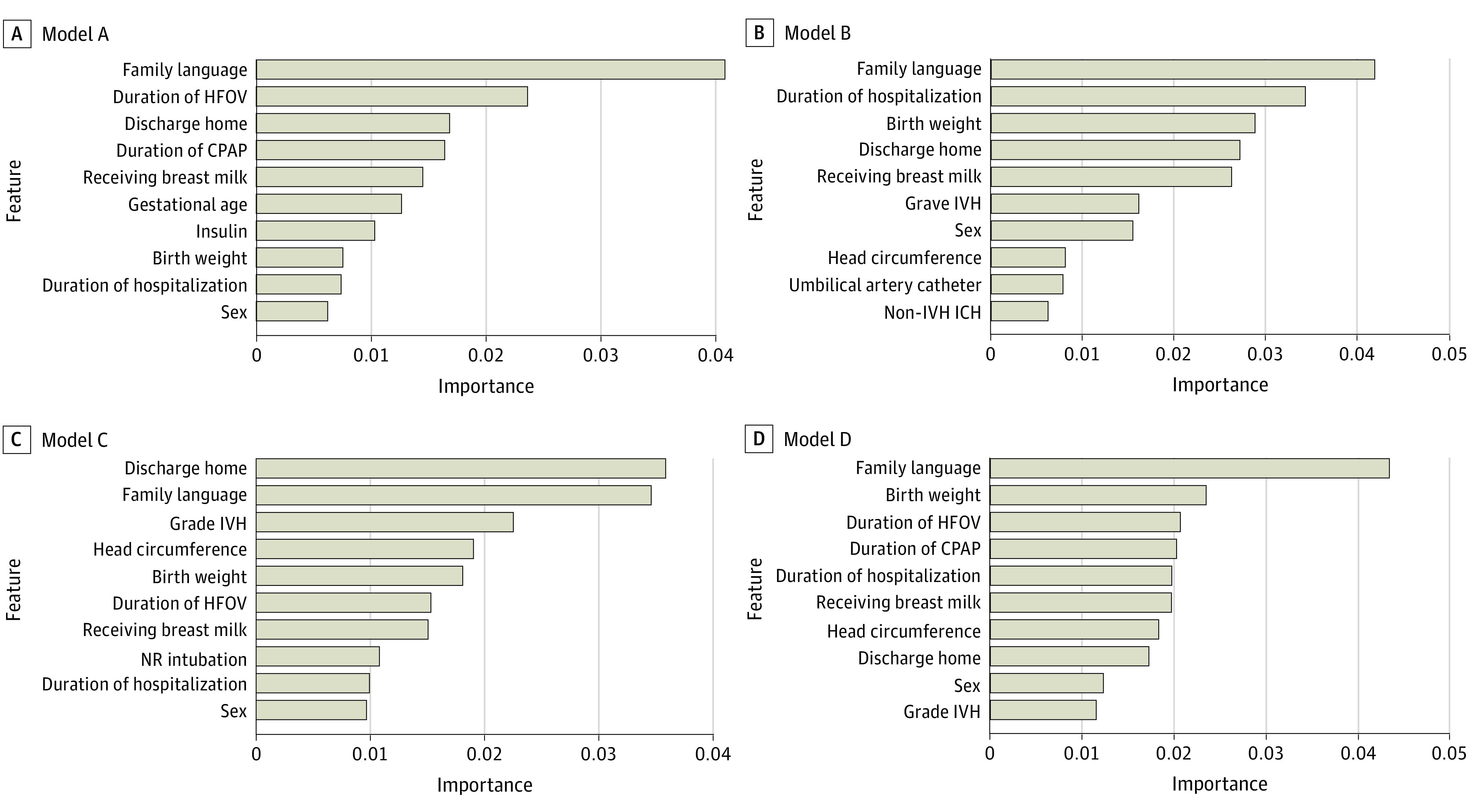
Feature Importance Plots for Models A to D Infants not discharged home were discharged to another hospital or unit or to home care. CPAP indicates continuous positive airway pressure; HFOV, high-frequency oscillatory ventilation; ICH, intracranial hemorrhage; IVH, intraventricular hemorrhage; NR, neonatal resuscitation.

Feature importance plots for models A to D are shown in Figure 2. The 10 most important features for prediction in model B were (listed in order of importance): family language, duration of hospitalization, birth weight, whether infant was discharged to home, whether infant was receiving breastmilk on discharge, grade of IVH, sex, head circumference, use of an umbilical artery catheter, and non-IVH intracranial hemorrhage. Family language, birth weight, sex, duration of hospitalization, whether infant was receiving breastmilk at discharge, and whether the infant was discharged directly home were included among the 10 most important features in all models. The 5 most important features for predicting cognitive delay in model B were non-Scandinavian family language, prolonged duration of hospitalization, low birth weight, discharge to other destination than home, and the infant not receiving breastmilk on discharge. Feature effect plots are contained in eFigures 4 and 5 in Supplement 1.

## Discussion

Using clinical data that are easily available, we have shown that a predictive model containing 26 features could already at discharge from the neonatal unit identify 93% of surviving VPT infants who would screen positive for CD at 24 months, with a corresponding specificity of 46%. Such a model could be incorporated into the electronic health record of the unit to allow alerts for the requirement of early targeted intervention.

Early intervention can improve the cognitive outcome of a preterm infant by approximately half of an SD on a standardized cognitive test at preschool age (equivalent to 7.5 IQ points).^14^ For 25% of infants with CD in this study, a half an SD improvement on the BSID-III would bring their cognitive function into the normal range, comparable with that of their typically developing peers. Alternatively, if these children are not identified at birth, they may pass early developmental checks^34^ and miss the window of opportunity for early intervention. Their cognitive difficulties may not come to light until they present with academic failure or behavioral difficulties in early school years.^35^

At the proposed decision threshold, a specificity of 46% would result in a substantial proportion of false-positive screening results. However, the acceptability of false-positive results among families is likely to be high given that all screening-positive infants are offered effective intervention and that the estimated number to treat to avoid 1 individual with cognition below the normal range is low (n = 12). Evidence-based programs enhancing the parent-infant relationship and enriching the early environment extend their benefits to domains other than cognition, such as improved infant motor and social skills and reduced parental anxiety and depression.^14,36,37,38^ Accordingly, any overtreatment could still be considered cost-effective.

The statistical approach used in this study was designed to optimize prediction, not investigate causal relationships. Important predictors, such as intubation at birth, use of umbilical catheters, insulin treatment for hyperglycemia, durations of ventilatory support and hospitalization, and whether the infant was receiving breastmilk, are likely to reflect severity of illness after birth. However, these factors are modifiable, and it cannot be excluded that the duration of time spent in the neonatal setting, where invasive procedures, artificial ventilation, and other noxious sensory exposures regularly occur, may have an effect on later cognitive outcomes.^39^ Developmental care, designed to adjust the neonatal environment to reduce stress and promote neural growth, may be beneficial,^40^ as may efforts to reduce duration of invasive ventilation and facilitate discharge to home. Early discharge for stable preterm infants has demonstrated both safety and improved parental well-being.^41,42^

### Limitations

Our study has important limitations. The study population consisted of survivors with a registered BSID-III cognitive score at 2 years of age. This population does not represent all VPT infants in Sweden. Although the follow-up program was launched in 2015, the completeness of BSID-III assessments has been limited by organizational and resource constraints, particularly by lack of testing capacity in remote areas. Most of the included children were born extremely preterm (<28 weeks) and resided in the 3 more densely populated urban areas.^43^ Selection biases may therefore have introduced an overestimation of CD rates in VPT infants, and the results may not be valid for all VPT children. However, extremely preterm infants assessed with the BSID-III in 2016 to 2019 did not differ significantly from those nonassessed with regard to sex, GA, birth weight, small for gestational age status, or incidence of IVH stages 3 to 4 in the neonatal period (M.N., unpublished data, November 1, 2023).

The outcome used in this study was cognitive function, but VPT infants often have deficits in multiple domains.^44^ Although individual cognitive trajectories will vary by familial and socioenvironmental factors, most extremely preterm survivors with low BSID-III scores at 2 years of age will have below-average IQ scores in later childhood.^45,46^ However, a significant proportion categorized as typically developed at 2 years of age will experience later worsening of cognitive function.^47^

Parental educational level and family language were important features in this study, and more detailed information on the socioeconomic and home environment may have improved model performance.^48^ Foreign family language may be a surrogate marker of low socioeconomic or immigration status. The administration of the BSID-III is heavily language dependent, and children taking the test in a nonnative language or through an interpreter may be disadvantaged, with apparent poor performance.^49^

## Conclusions

This prognostic study found that it is possible to identify CD before discharge from the neonatal unit in VPT infants. Future work should include further model validation on VPT infants. Other research groups using neurophysiological, microbiome, or imaging data should consider inclusion of the 26 clinical features identified herein in coming predictive models.

## Notes

Supplement 1. **eMethods.** Supplemental Methods **eFigure 1.** Feature Selection Using Boruta Algorithm **eFigure 2.** Features Selected by Boruta Algorithm With Correlation Coefficients >0.70 **eFigure 3.** Receiver Operating Characteristic Curves of Models A-D **eFigure 4.** Feature Effect Plots for Model B (Logistic Regression) **eFigure 5.** Feature Effect Plots for Model D (Gradient Boosted Machine) **eTable 1.** Features With >25% Missing Values **eTable 2.** Missing Data for 90 Features Considered in Modelling Process **eTable 3.** Characteristics of Study Population **eTable 4.** Final 26 Features Included in Model **eTable 5.** Results of Internal Validation Using Ten-Fold Cross Validation for Models A-D
eReferences

Supplement 2.
Data Sharing Statement
