# Supplementary material for: Prediction of 2-Year Cognitive Outcomes in Very Preterm Infants Using Machine Learning Methods
Source: JAMA Netw Open. 2023 Dec 26;6(12):e2349111. doi: 10.1001/jamanetworkopen.2023.49111 (PMC10751596; doi:10.1001/jamanetworkopen.2023.49111)
Supplement: Supplement 1. — eMethods. Supplemental Methods eFigure 1. Feature Selection Using Boruta Algorithm eFigure 2. Features Selected by Boruta Algorithm With Correlation Coefficients >0.70 eFigure 3. Receiver Operating Characteristic Curves of Models A-D eFigure 4. Feature Effect Plots for Model B (Logistic Regression) eFigure 5. Feature Effect Plots for Model D (Gradient Boosted Machine) eTable 1. Features With >25% Missing Values eTable 2. Missing Data for 90 Features Considered in Modelling Process eTable 3. Characteristics of Study Population eTable 4. Final 26 Features Included in Model eTable 5. Results of Internal Validation Using Ten-Fold Cross Validation for Models A-D eReferences [file jamanetwopen-e2349111-s001.pdf]

Supplementary Online Content

Bowe AK, Lightbody G, Staines A, Murray DM, Norman M. Prediction of 2-year coginitive outcomes in very preterm infants using machine learning methods. *JAMA Netw Open*. 2023;6(12):e2349111. doi:10.1001/jamanetworkopen.2023.49111

eMethods. Supplemental Methods
eFigure 1. Feature Selection Using Boruta Algorithm
eFigure 2. Features Selected by Boruta Algorithm With Correlation Coefficients >0.70
eFigure 3. Receiver Operating Characteristic Curves of Models A-D
eFigure 4. Feature Effect Plots for Model B (Logistic Regression)
eFigure 5. Feature Effect Plots for Model D (Gradient Boosted Machine)
eTable 1. Features With >25% Missing Values
eTable 2. Missing Data for 90 Features Considered in Modelling Process
eTable 3. Characteristics of Study Population
eTable 4. Final 26 Features Included in Model
eTable 5. Results of Internal Validation Using Ten-Fold Cross Validation for Models A-D
eReferences

This supplementary material has been provided by the authors to give readers additional information about their work.

© 2023 Bowe AK et al. *JAMA Network Open.*

eMethods. Supplemental Methods

Methodology

Rebalancing dataset

An imbalanced dataset is one where the classification categories are not approximately equally represented.[1] In registry data and cohort data this is often the case, where datasets are composed largely of those without the condition or disease of interest. Most machine learning algorithms assume a roughly balanced class distribution. When class imbalance is present the decision boundary is biased toward the majority class.[2] An algorithm which learns from class-imbalanced data tends to have poor predictive accuracy for samples in the minority class when it is shown new data. It tends to classify most new samples as being in the majority class.[1,3] In our original dataset there were n=231 infants with cognitive delay at 2 year follow up and n= 831 infants without delay. Training the algorithms on this class imbalanced data would likely result in a classifier bias towards the majority class (i.e. typical cognitive development). To address this, the synthetic minority oversampling technique (SMOTE) was applied to the training dataset.[1]

The aim of the SMOTE algorithm is to create a balanced dataset where the outcome classes, in this case 'cognitive delay' and 'typical cognitive development', have approximately equal observations. It achieves this through a combination of oversampling the minority class and undersampling the majority class. Unlike other oversampling techniques which simply duplicate minority class cases, SMOTE creates synthetic examples in the minority class.[1] Each minority class sample can be considered as operating in a 'feature space'.[1] The algorithm randomly selects *k* minority class samples from a sample's nearest minority class neighbours (*k* is a parameter set by the user). Then, synthetic examples are created at random locations along the lines joining the *k* minority class neighbours. This is repeated until the desired amount of oversampling, set by the user using the 'perc.over' parameter, is achieved. In our study, oversampling was set to perc.over = 200 and the number of nearest neighbours used to generate new samples was set to *k*=5. For each existing sample categorised as 'cognitive delay' in the training dataset (n=162) two new samples were generated using information from the 5 nearest neighbours of each 'cognitive delay' sample, resulting in n=486 samples categorised as 'cognitive delay' in the rebalanced dataset.[1,4] Undersampling of the majority class, if required, is achieved by random removal of samples from the majority class. In our study SMOTE was applied only to the training dataset. This ensured that the final model was tested on completely unseen and untouched real life data.

Feature Effect

Feature effect was explored using partial dependence plots (PDPs) created with the 'iml' package.[5] The PDPs shows the average predicted probability of cognitive delay (CD) for a given feature value on the x-axis. For every value *x* of the feature of interest, model predictions are calculated and averaged across every observation, based on the observation having *x* as the value for that feature.[5] A lower value on the y-axis suggests that CD is less likely at that value of the feature on x-axis. Conversely, a higher value on the y-axis suggests a higher probability of CD. PDPs ignore whether the value *x* is plausible or even possible for that observation, therefore making plot estimations unreliable in the presence of correlated features. Plots should also be interpreted with caution for values with few or no observations. The feature effect for the ten most important features in Model B and Model D are shown in eFigure 1 and eFigure 2. In Model B head circumference was strongly correlated with gestational age (Pearson correlation coefficient (r) = 0.80), birthweight (r = 0.82) and duration of hospitalisation (r = -0.63). Therefore, for the plot showing the effect of head circumference, the correlated features were removed from the model. Similarly, the plot for birthweight excludes the effect of gestational age (r = 0.71) and head circumference (r=0.82). The same procedure was performed for correlated features in Model D. The PDPs show how a machine learning model, such as the gradient boosted machine model in eFigure 3, can model non-linear relationships.

© 2023 Bowe AK et al. *JAMA Network Open.*

eFigure 1. Feature selection using Boruta algorithm

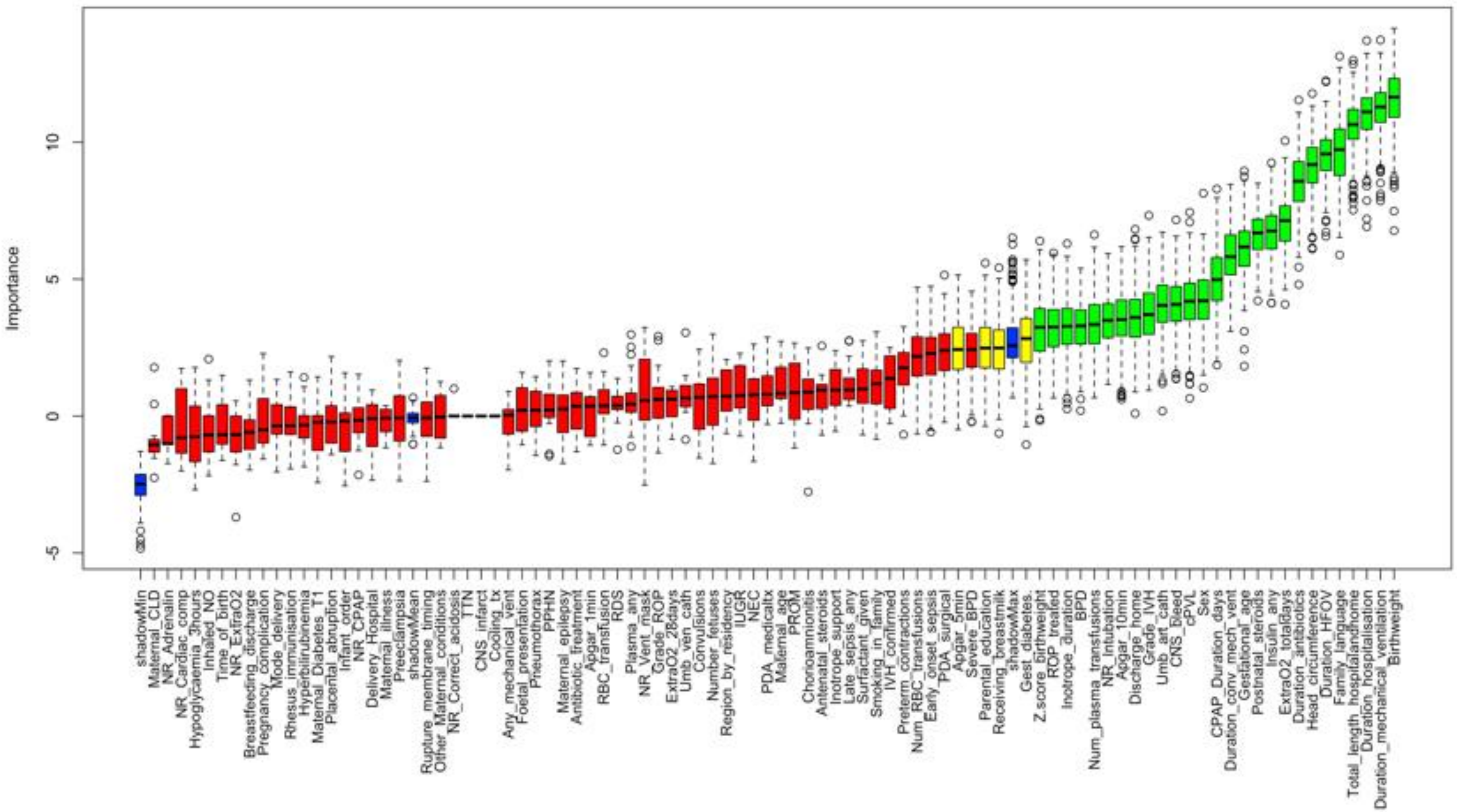

eFigure 1 Legend. CLD – Maternal chronic lung disease, NR – Neonatal resuscitation, cardiac_comp – cardiac compressions, CPAP – Continuous positive airway pressure, TTN – Transient tachypnoea of the newborn, CNS – Central nervous system, vent – ventilation, PPHN – Persistent pulmonary hypertension of the newborn, RBC – Red blood cell, RDS – Respiratory distress syndrome, ROP – Retinopathy of prematurity, Umb_ven_cath – Umbilical venous catheter, Umb_art_cath – Umbilical arterial catheter, IUGR – Intrauterine growth restriction, NEC – Necrotising enterocolitis, PDA – Patent ductus arteriosus, PROM – Preterm rupture of membranes, IVH – Intraventricular hemorrhage, BPD – Bronchopulmonary dysplasia, Gest_diabetes – Gestational diabetes, cPVL – Cystic periventricular leukomalacia, Duration_conv_mech_vent – Duration of conventional mechanical ventilation, HFOV – High frequency oscillatory ventilation.
A standardized feature importance score is shown in the y-axis. The blue boxplots represent the minimum, mean, and maximum z-scores of the shadow attributes which are created by randomly shuffling the values of the original features. The green, yellow, and red boxplots represent the z-scores of the confirmed, tentative, and rejected features respectively.

© 2023 Bowe AK et al. *JAMA Network Open.*

eFigure 2. Features selected by Boruta algorithm with correlation coefficients >0.70

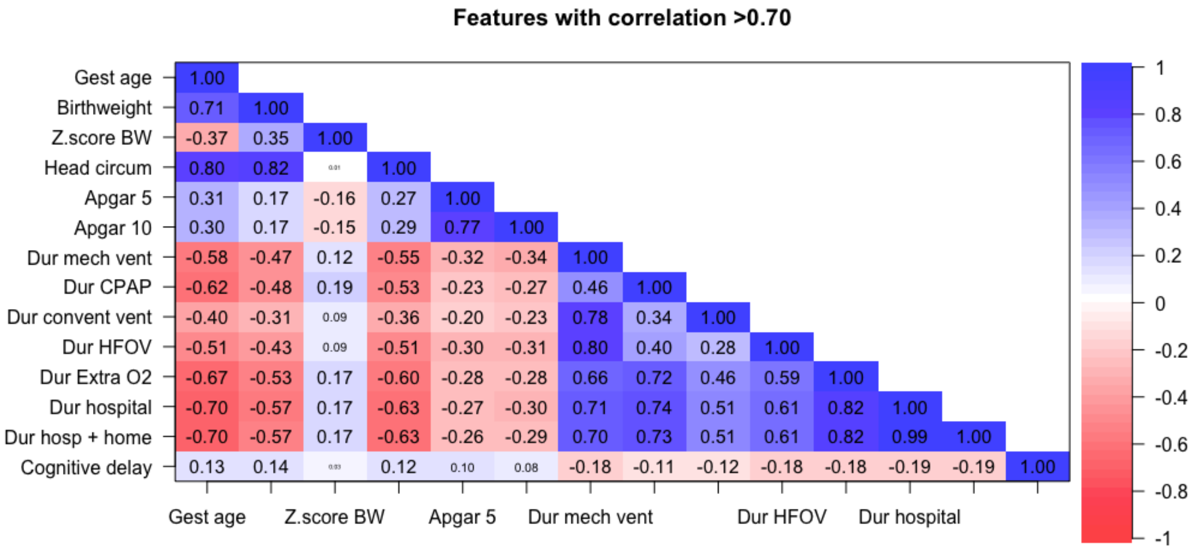

eFigure 2 Legend. Gest age – Gestational age, Z.score BW – Z score for birthweight, Head circum – Head circumference, Dur mech vent – Duration of all mechanical ventilation in days, Dur CPAP – Duration of continuous positive airway pressure in days, Dur convent vent – Duration of conventional mechanical ventilation in days, Dur HFOV – Duration of high frequency oscillatory ventilation in days, Dur Extra O2 – Duration of extra oxygen, Dur hospital – Duration of hospitalisation, Dur hosp + home – Duration of hospital and home care

© 2023 Bowe AK et al. *JAMA Network Open.*

eFigure 3. Receiver operating characteristic curves of Models A-D

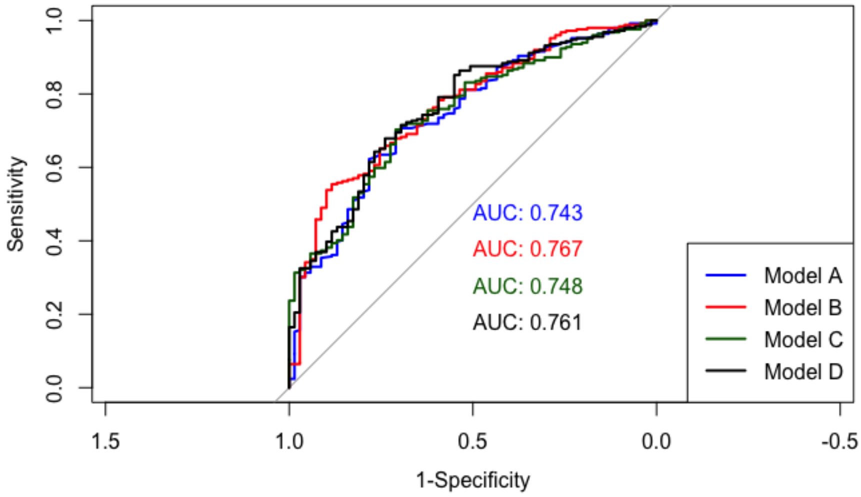

eFigure 3 Legend: The receiver operating characteristic (ROC) curves for Models A-D tested on the unseen dataset are shown. The sensitivity is shown on the y-axis and 1-Specificity on the x-axis.
To ensure the performance of Model B (selected as our optimal model) was not affected by bias introduced by a single 70-30 split of the dataset, we performed ten random simulations of the 70-30 split and tested the AUROC across each unseen dataset. The mean AUROC of Model B across the ten simulations of the 70-30 split was 0.76 (95% Confidence Interval 0.69 – 0.82).

© 2023 Bowe AK et al. *JAMA Network Open.*

eFigure 4. Feature effect plots for Model B (Logistic Regression)

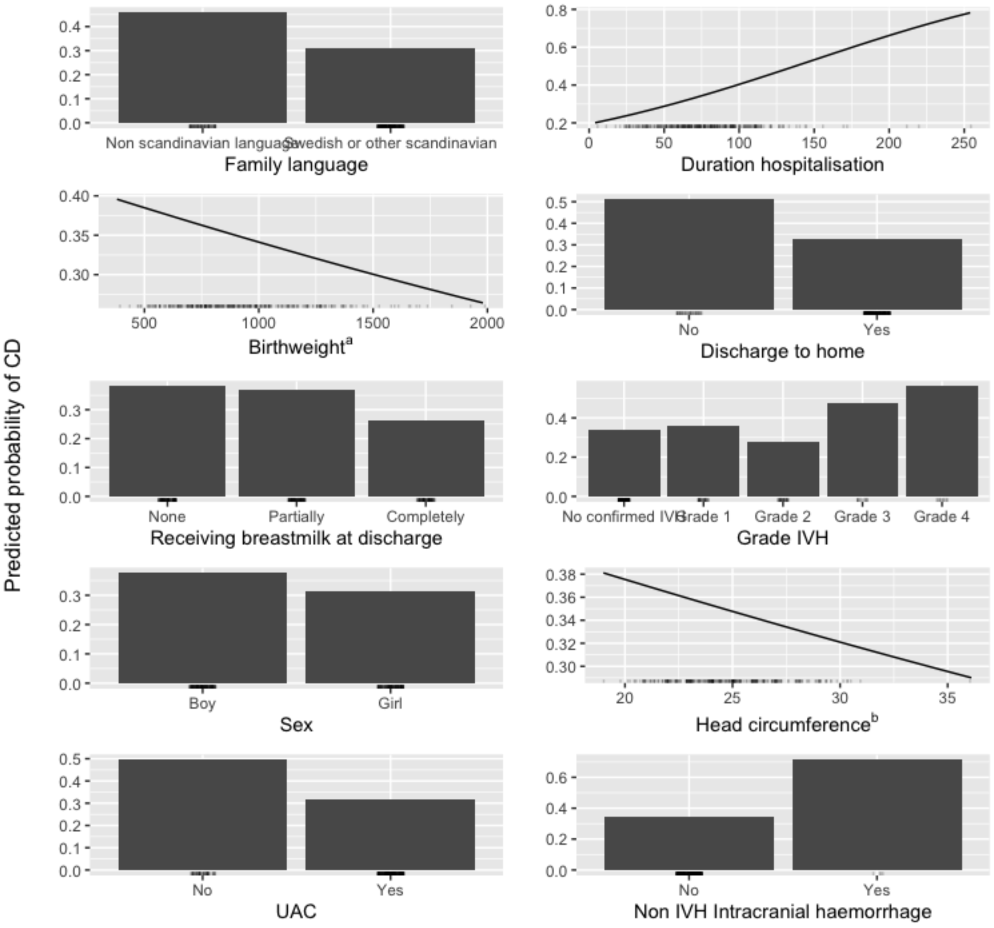

eFigure 4 Legend: [a] Model excludes gestational age and head circumference. [b] Model excludes gestational age, birthweight and duration of hospitalisation. On the y-axis of the plots is the probability of cognitive delay and on the x-axis is either the categorical feature label or the continuous feature value. The distribution of feature values is shown along the x-axis. The predicted probability of CD was higher for families with who reported a non-Scandinavian family language, for those who were not discharged to home, for boys, for those who received no breastmilk at discharge, for those with Grade 4 IVH and for those with Non IVH intracranial haemorrhage. As the duration of hospitalisation increased, the predicted probability of CD increased. As head circumference and birthweight increased, the predicted probability of CD decreased.

© 2023 Bowe AK et al. *JAMA Network Open.*

eFigure 5. Feature effect plots for Model D (Gradient Boosted Machine)

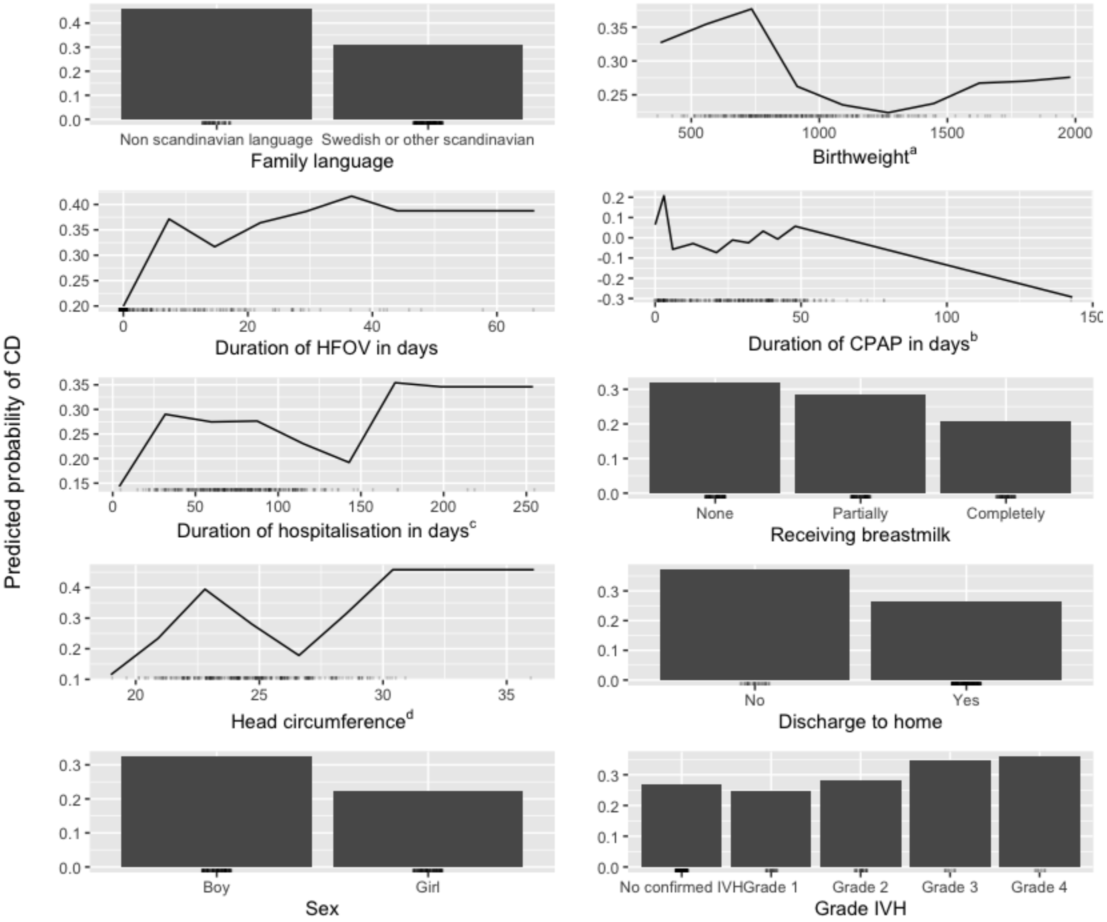

eFigure 5 Legend: [a] Model excludes gestational age and head circumference, [b] Model excludes duration of hospitalisation, [c] Model excludes Duration of CPAP in days, [d] Model excludes birthweight and gestational age. On the y-axis of the plots is the probability of cognitive delay and on the x-axis is either the categorical feature label or the continuous feature value. The distribution of feature values is shown along the x-axis. For plots examining continuous features, for values where there are few or no observations the plots should be interpreted with caution as estimates may be unreliable.

© 2023 Bowe AK et al. *JAMA Network Open.*

eTable 1. Features with >25% missing values

Feature	Missing n (%)
Umbilical artery P02	648 (61.0%)
Umbilical artery PCO2	621 (58.5%)
Umbilical artery pH	608 (57.3%)
Venous P02	576 (54.2%)
Venous PC02	534 (50.3%)
Venous pH	521 (49.1%)
Birth length	269 (25.3%)

© 2023 Bowe AK et al. *JAMA Network Open.*

eTable 2. Missing data for 90 features considered in modelling process

Feature	Description	Feature type	N (%) missing
Parent education[a]	0-3 years<br>3-6 years<br>7-9 years<br>10-12 years<br>>12 years	Categorical	203 (19.1)
Head circumference	Measured in cm	Numeric	179 (16.9)
Rupture of membrane timing[a]	0-12 hours<br>12-24 hours<br>>24 hours – 1 week<br>>1 week – 4 weeks	Categorical	178 (16.8)
Receiving breastmilk at discharge	Completely<br>Partially<br>None	Categorical	149 (14.0)
Family language	Swedish or other Scandinavian language<br>Non Scandinavian language	Categorical	148 (14.0)
Foetal presentation	Breech<br>Cephalic<br>Transverse<br>Other	Categorical	117 (11.0)
Maximum grade of retinopathy of prematurity (ROP)	No ROP<br>Grade 1<br>Grade 2<br>Grade 3<br>Grade 4<br>Grade 5	Categorical variable	62 (5.8)
ROP treated	Yes<br>No	Categorical	62 (5.8)
Antenatal steroids[a]	No<br>Yes, <8 hours before delivery<br>Yes, 8-24 hours before delivery<br>Yes, >24 hours before delivery<br>Yes, missing time data	Categorical	61 (5.7)
Apgar 1 minute		Numeric	34 (3.2)
Apgar 10 minutes		Numeric	29 (2.7)
Number of erythrocyte transfusions		Numeric	28 (2.6)
Apgar 5 minutes		Numeric	26 (2.4)
Duration of continuous positive airway pressure (CPAP)	Duration in days	Numeric	24 (2.3)
Highest confirmed grade of intraventricular haemorrhage (IVH)	No confirmed IVH<br>Grade 1<br>Grade 2<br>Grade 3<br>Grade 4	Categorical (derived)	17 (1.6)
Number of plasma transfusions		Numeric	12 (1.1)
Mode of delivery	Elective c-section<br>Emergency c-section<br>Vacuum<br>Vaginal	Categorical (derived)	10 (0.9)

© 2023 Bowe AK et al. *JAMA Network Open.*

Cardiac compressions	Yes<br>No	Categorical	9 (0.8)
CPAP during resuscitation	Yes<br>No	Categorical	9 (0.8)
Ventilation mask during resuscitation	Yes<br>No	Categorical	9 (0.8)
Adrenaline during resuscitation	Yes<br>No	Categorical	9 (0.8)
Correction of acidosis during resuscitation	Yes<br>No	Categorical	9 (0.8)
Intubation during resuscitation	Yes<br>No	Categorical	9 (0.8)
Maternal illness	Yes<br>No	Categorical	7 (0.7)
Pregnancy complication	Yes<br>No	Categorical	7 (0.7)
Placental abruption	Yes<br>No	Categorical	7 (0.7)
Chorioamnionitis	Yes<br>No	Categorical	7 (0.7)
Preterm contractions	Yes<br>No	Categorical	7 (0.7)
Intrauterine growth restriction	Yes<br>No	Categorical	7 (0.7)
Preeclampsia	Yes<br>No	Categorical	7 (0.7)
Rupture of membranes before 37 weeks	Yes<br>No	Categorical	7 (0.7)
Rhesus immunisation	Yes<br>No	Categorical	7 (0.7)
Birthweight	Birthweight in grams (20g intervals)	Numeric	7 (0.6)
Z.score for birthweight	Gestational-age specific birthweight z-score	Numeric	6 (0.6)
Maternal Epilepsy	Yes<br>No	Categorical	5 (0.5)
Maternal type 1 diabetes	Yes<br>No	Categorical	5 (0.5)
Maternal chronic lung disease or asthma	Yes<br>No	Categorical	5 (0.5)
Maternal age	Age in years	Numeric	1 (0.09)

Categorizations of parental education, timing of rupture of membranes, and timing of antenatal corticosteroids reflect how these variables were reported to the Swedish Neonatal Quality Register.

© 2023 Bowe AK et al. *JAMA Network Open.*

eTable 3. Characteristics of study population

Variable	Valid n	Total n (%)	Typical cognitive development n = 831	Cognitive delay n = 231	P-value
**Pregnancy**					
No. of foetuses	1,062				
Singleton		751 (70.7)	589 (70.9)	162 (70.1)	
Twin		286 (26.9)	222 (26.7)	64 (27.7)	
Triplet		25 (2.4)	20 (2.4)	5 (2.2)	0.898[a]
Maternal illness	1,055	459 (43.5)	351 (42.5)	108 (47.0)	0.264[b]
Epilepsy	1,057	8 (0.8)	7 (0.8)	1 (0.4)	1.000[a]
Type 1 DM	1,057	6 (0.6)	4 (0.5)	2 (0.9)	0.616[a]
Chronic lung or asthma	1,057	51 (4.8)	42 (5.1)	9 (3.9)	0.578[b]
Other	1,057	348 (32.9)	264 (31.9)	84 (36.5)	0.217[b]
Pregnancy complication	1,055	1,003 (95.1)	778 (94.3)	225 (97.8)	0.023[a]
Placental abruption	1,055	148 (14.0)	115 (13.9)	33 (14.3)	0.960[b]
Chorioamnionitis	1,055	77 (7.3)	53 (6.4)	24 (10.4)	0.054[b]
Gestational diabetes	1,055	14 (1.3)	8 (1.0)	6 (2.6)	0.094[a]
IUGR	1,055	178 (16.9)	145 (17.6)	33 (14.3)	0.291[b]
Preeclampsia	1,055	161 (15.3)	125 (15.2)	36 (15.7)	0.153[b]
ROM <37 weeks (no contractions)	1,055	169 (16.0)	118 (14.3)	51 (22.2)	0.005[b]
Antenatal steroids	1,001				
No		67 (6.7)	50 (6.4)	17 (7.8)	
< 8 hours before delivery		148 (14.8)	123 (15.7)	25 (11.4)	
8-24 hours before delivery		152 (15.2)	119 (15.2)	33 (15.1)	
>24 hours before delivery		617 (61.6)	478 (61.1)	139 (63.5)	
Yes, timing unknown		17 (1.7)	12 (1.5)	5 (2.3)	0.461[a]
**Birth**					
Delivery hospital	1,062				
Level 1/2		186 (17.5)	154 (18.5)	32 (13.9)	
Level 3/4		871 (82.0)	673 (81.0)	198 (85.7)	
Other		5 (0.5)	4 (0.5)	1 (0.4)	0.251[a]
Time of birth	1,062				
8am-5pm		552 (52.0)	426 (51.3)	126 (54.5)	
5pm-8am		510 (48.0)	405 (48.7)	105 (45.5)	0.419[b]
Presentation	945				
Breech		288 (30.5)	227 (30.8)	61 (29.3)	
Cephalic		565 (59.8)	439 (59.6)	126 (60.6)	
Transverse		38 (4.0)	41 (5.6)	13 (6.2)	
Other		54 (5.7)	30 (4.1)	8 (3.8)	0.957[a]
Maternal age at delivery	1,061				
N, mean (sd)		31.8 (5.6)	831, 31.4 (5.5)	230, 31.2 (5.8)	0.739[c]
Delivery	1,052				
Forceps or vacuum		5 (0.5)	5 (0.6)	0 (0.0)	
Elective Section		29 (2.8)	24 (2.9)	5 (2.2)	
Emergency Section		679 (64.5)	524 (63.8)	155 (67.1)	
Vaginal		339 (32.2)	268 (32.6)	71 (30.7)	0.675[a]
Gestational age	1,062				
N, mean (sd)		26.5 (2.2)	831, 26.7 (2.2)	231, 26.0 (2.2)	<0.001[c]
Birthweight - g	1,055				
N, median (IQR)		880 (380)	824, 900 (365)	231, 800 (320)	<0.001[d]
Z-score for birthweight	1,054				
N, median (IQR)		-1.06 (2.00)	825, -0.99 (1.98)	231, -1.19 (2.06)	0.157[d]
Head circumference	883				
N, mean (sd)		24.7 (2.5)	685, 24.9 (2.5)	198, 24.2 (2.5)	0.001[c]
Sex	1,062				
Boy		566 (53.3)	414 (49.8)	152 (65.8)	
Girl		496 (46.7)	417 (50.2)	79 (34.2)	<0.001[b]
Apgar 1 min	1,028				

© 2023 Bowe AK et al. *JAMA Network Open.*

N, median (IQR)		6 (4)	805, 6 (4)	223, 5 (3.5)	0.013[d]
Apgar 5 min	1,036				
N, median (IQR)		8 (3)	809, 8 (3)	227, 7 (4)	0.001[d]
Apgar 10 min	1,033				
Median (IQR)		9 (2)	808, 9 (2)	225, 9 (3)	0.010[d]
**Neonatal Resuscitation**					
Extra oxygen	1,053	835 (79.3)	650 (78.7)	185 (81.5)	0.406[b]
CPAP	1,053	777 (73.8)	621 (75.2)	156 (68.7)	0.061[b]
Chest compressions	1,053	11 (1.0)	10 (1.2)	1 (0.4)	0.473[a]
Adrenaline	1,053	3 (0.3)	2 (0.2)	1 (0.4)	0.518[a]
Ventilation mask	1,053	683 (64.9)	518 (62.7)	165 (72.7)	0.007[b]
Correction acidosis	1,053	4 (0.4)	4 (0.5)	0 (0.0)	0.583[a]
Intubation	1,053	371 (35.2)	265 (32.1)	106 (46.7)	<0.001[b]
**Respiratory**					
TTN any	1,062	1 (0.1)	1 (0.1)	0 (0.0)	1.000[a]
RDS	1,062	887 (83.5)	680 (81.8)	207 (89.6)	0.007[b]
Pneumothorax	1,062	33 (3.1)	23 (2.8)	10 (4.3)	0.320[b]
BPD[e]	1,062	637 (60.0)	475 (57.2)	162 (70.1)	<0.001[b]
Severe BPD	1,062	106 (10.0)	71 (8.5)	35 (15.2)	0.005[b]
PPHN	1,062	81 (7.6)	56 (6.7)	25 (10.8)	0.054[b]
Extra O2 total days	1,062				
Median (IQR)		49 (65)	831, 45 (62)	231, 67 (65)	<0.001[d]
Surfactant given	1,062	697 (65.6)	530 (63.8)	167 (72.3)	0.020[b]
Any mechanical ventilation	1,062	690 (65.0)	556 (62.5)	134 (77.5)	<0.001[b]
Days mechanical vent total	1,062				
N, Median (IQR)		4 (17)	831, 3 (14)	231, 12 (26)	<0.001[d]
Days CPAP	1,038				
N, median (IQR)		26 (30)	809, 25 (30)	229, 30 (30)	<0.001[d]
Days conventional mechanical ventilation	1,062				
N, median (IQR)		1 (6)	831, 1 (5)	231, 3 (9)	<0.001[d]
Days HFOV	1,062				
N, median (IQR)		0 (8)	831, 0 (5)	231, 3 (15)	<0.001[d]
Inhaled NO	1,062	45 (4.2)	29 (3.5)	16 (6.9)	0.035[b]
Postnatal steroids	1,062	341 (32.1)	238 (28.6)	103 (44.6)	<0.001[b]
**Infection**					
Early onset sepsis	1,062	14 (1.3)	6 (0.7)	8 (3.5)	0.004[a]
Late onset sepsis	1,062	206 (19.4)	151 (18.2)	55 (23.8)	0.068[b]
Antibiotic treatment	1,062	979 (92.2)	762 (91.7)	217 (93.9)	0.325[a]
Days of antibiotics	1,062				
N, median (IQR)		14 (20)	831, 12 (19)	231, 20 (25)	<0.001[d]
**Neurology**					
Non IVH intracranial haemorrhage	1,062	12 (1.1)	6 (0.7)	6 (2.6)	0.028[a]
Seizures	1,062	16 (1.5)	9 (1.1)	7 (3.0)	0.059[a]
EEG or aEGG	1,062	69 (6.5)	49 (5.9)	20 (8.7)	0.175[b]
PVL - any	1,062	17 (1.6)	8 (1.0)	9 (3.9)	0.004[a]
IVH - confirmed	1,045	300 (28.7)	217 (26.5)	83 (36.7)	0.001[b]
<u>Highest grade IVH</u>	1,045				
No confirmed IVH		745 (71.3)	602 (73.5)	143 (63.3)	
Grade 1		129 (12.3)	100 (12.2)	29 (12.8)	
Grade 2		97 (9.3)	73 (8.9)	24 (10.6)	
Grade 3		36 (3.4)	24 (2.9)	12 (5.3)	
Grade 4		38 (3.6)	20 (2.4)	18 (8.0)	<0.001[b]
**Other diseases**					
Hyperbilirubinemia	1,062	946 (89.1)	738 (88.8)	208 (90.0)	0.680[b]
NEC	1,062	78 (7.3)	53 (6.4)	25 (10.8)	0.032[b]
PDA – medical tx	1,062	388 (36.5)	288 (34.7)	100 (43.3)	0.020[b]
PDA – surgical tx	1,062	153 (14.4)	104 (12.5)	49 (21.2)	0.001[b]
<u>Max Grade ROP</u>					
None	1,052	542 (51.5)	444 (54.1)	98 (42.4)	

© 2023 Bowe AK et al. *JAMA Network Open.*

Grade 1		142 (13.5)	115 (14.0)	27 (11.7)	
Grade 2		167 (15.9)	125 (15.2)	42 (18.2)	
Grade 3		198 (18.8)	136 (16.6)	62 (26.8)	
Grade 4		3 (0.3)	1 (0.1)	2 (0.9)	<0.001[a]
**Other treatments**					
Insulin	1,062	131 (12.3)	88 (10.6)	43 (18.6)	0.002[b]
ROP treated	1,000	124 (12.4)	84 (10.8)	40 (17.9)	0.007[b]
Inotrope support	1,062	200 (18.8)	136 (16.4)	64 (27.7)	<0.001[b]
Inotrope duration	1,062				
N, median (IQR)		0 (0)	831, 0 (0)	231, 0 (1.5)	<0.001[d]
Any erythrocyte transfusion	1,062	911 (85.8)	705 (84.8)	206 (89.2)	0.118[b]
Erythrocyte number transfusions	1,034				
N, median (IQR)		13 (28)	808, 13 (27)	226, 14 (26)	0.034[d]
Any plasma transfusion	1,062	548 (51.6)	408 (49.1)	140 (60.6)	<0.001[b]
Plasma number transfusion	1,050				
M, median (IQR)		2 (11)	822, 1 (11)	228, 2 (19)	<0.001[d]
**Hospital stay and discharge**					
Length hospital stay	1,062				
N, median (IQR)		2 (11)	831, 72 (40)	231, 83 (42.5)	<0.001[d]
Length hospital and homecare	1,062				
N, median (IQR)		75 (40)	831, 73 (40)	231, 85 (43.5)	<0.001[d]
Discharged to home	1,062	927 (87.3)	746 (89.8)	181 (78.4)	<0.001[b]
Breastfeeding on discharge	942				
None		421 (44.7)	311 (41.9)	110 (55.3)	
Partial		454 (48.2)	373 (50.2)	81 (40.7)	
Completely		67 (7.1)	59 (7.9)	8 (4.0)	0.002[a]
Receiving breastmilk on discharge	913				
None		320 (35.0)	235 (32.2)	85 (46.2)	
Partially		355 (38.9)	284 (39.0)	71 (38.6)	
Completely		238 (26.1)	210 (28.8)	28 (15.2)	<0.001[b]
**Sociodemographic**					
Family language	914				
Swedish or other Scandinavian language		641 (70.1)	541 (74.8)	100 (52.5)	<0.001[b]
Smoking in family	1,062	131 (12.3)	98 (11.8)	33 (14.3)	0.365[b]
Parental education					
0-3 years		8 (0.9)	5 (0.7)	3 (1.6)	
3-6 years		3 (0.3)	2 (0.3)	1 (0.5)	
7-9 years		50 (5.8)	31 (4.6)	19 (10.4)	
10-12 years		322 (37.5)	243 (35.9)	79 (43.2)	
>12 years		476 (55.4)	395 (58.4)	81 (44.3)	<0.001[a]
**Bayleys Scales**					
Corrected age at Bayleys III (years)	1,024				
N, mean (sd)		2.2 (0.2)	804, 2.2 (0.2)	220, 2.2 (0.2)	0.593[c]
Cognition index score	1,062				
N, mean (sd)		96.8 (16.1)	831, 103 (10.5)	231, 73.5 (10.0)	<0.001[c]

[a] Fishers exact test, [b] Pearson's Chi-squared test, [c] Welch two sample t test,
[d] Wilcoxan rank sum test
[e] Defined as a registered ICD-10 code for BPD (P27.1) or a registration of supplemental oxygen use at 36 weeks of postmenstrual age
[f] Reasons for not completing: Child declined or would not participant n=25, Inattention, hyperactivity or fatigue n=7, Language barrier n=1, Parent declined n=5, Resources or administrative reason n=6, child unable to complete n=14, reason unclear or not recorded n=6.

© 2023 Bowe AK et al. *JAMA Network Open.*

eTable 4. Final 26 features included in model

1	Gestational diabetes
2	Gestational age
3	Birthweight
4	Head circumference
5	Sex
6	Apgar 10 minutes
7	Intubation during neonatal resuscitation
8	Umbilical artery catheter
9	Bronchopulmonary dysplasia
10	Duration of CPAP (days)
11	Duration of conventional mechanical ventilation (days)
12	Duration of high frequency oscillatory ventilation (days)
13	Postnatal steroids
14	Duration antibiotics (days)
15	CNS bleed (excluding IVH)
16	Cystic perventricular leukomalacia (cPVL)
17	Grade IVH
18	Insulin treatment
19	Retinopathy of prematurity treated
20	Duration of inotropes (days)
21	Number of plasma transfusions
22	Duration of hospitalisation (days)
23	Discharge to home
24	Receiving breastmilk
25	Family language
26	Parental education

© 2023 Bowe AK et al. *JAMA Network Open.*

eTable 5. Results of internal validation using ten-fold cross validation for Models A-D

Model Name	Algorithm	Features	Hyperparameters tuned in grid search (values)	Accuracy
Model A	Random forest	26	Mtry (1:26)	0.86
Model B	Logistic Regression	26		0.69
Model C	Support vector machine	26	C (0.25,0.50,1,2,4,8,16,32,64,128)[a] Sigma (0.01, 0.02, 0.05, 0.1, 0.2, 0.3, 0.4)	0.82
Model D	Gradient boosting machine	26	Ntrees (400,500,600,700,800,900)[a]<br>Interaction depth (4,5,6,7,8,9,10,11,12,13,14,15)<br>Shrinkage (0.1)<br>n.minobsinnode (10)	0.87

[a] An initial random gridsearch was performed first to inform the range of tuning parameters chosen for training process.

© 2023 Bowe AK et al. *JAMA Network Open.*

eReferences

1 Chawla N, Bowyer K, Hall L, Kegelmeyer W. SMOTE: synthetic minority over-sampling technique. *J Artif Intell Res* 2002; 16: 341–348.

2 Kim A, Jung I. Optimal selection of resampling methods for imbalanced data with high complexity. PLoS One. 2023 Jul 27;18(7):e0288540. doi: 10.1371/journal.pone.0288540. PMID: 37498823; PMCID: PMC10374143.

3 Blagus R, Lusa L. SMOTE for high-dimensional class-imbalanced data. *BMC Bioinformatics* 2013; 14: 106.

4 RDocumentation. SMOTE: SMOTE algorithm for unbalanced classification problems.[Internet] [cited 9th November 2023]. Available from https://www.rdocumentation.org/packages/DMwR/versions/0.4.1/topics/SMOTE

5 Molnar C, Bischl B, Casalicchio G. Iml: An R package for Interpretable Machine Learning. *The Journal of Open Source Software* 2018; 3: 786.

© 2023 Bowe AK et al. *JAMA Network Open.*
