# Supplementary material for: Prediction of 2-Year Cognitive Outcomes in Very Preterm Infants Using Machine Learning Methods
Source: JAMA Netw Open. 2023 Dec 26;6(12):e2349111. doi: 10.1001/jamanetworkopen.2023.49111 (PMC10751596; doi:10.1001/jamanetworkopen.2023.49111)
Supplement: Supplement 2. — Data Sharing Statement [file jamanetwopen-e2349111-s002.pdf]

Data Sharing Statement

Bowe. Prediction of 2-Year Cognitive Outcomes in Very Preterm Infants Using Machine Learning Methods. *JAMA Netw Open*. Published December 26, 2023. doi:10.1001/jamanetworkopen.2023.49111

Data
Data available: No
